# The Efficacy and Safety of Pimecrolimus in Patients With Facial Seborrheic Dermatitis: A Systematic Review of Randomized Controlled Trials

**DOI:** 10.7759/cureus.27622

**Published:** 2022-08-02

**Authors:** Odeh Alsmeirat, Som Lakhani, Musab Egaimi, Osama Idris, Mohamed Elkhalifa

**Affiliations:** 1 Internal Medicine, Sheikh Khalifa Specialty Hospital, Ras Al Khaimah, ARE; 2 Dermatology, Parul University, Vadodara, IND

**Keywords:** skin condition, seborrheic eczema, calcineurin inhibitors, topical, pimecrolimus, facial seborrheic dermatitis

## Abstract

Facial seborrheic dermatitis (SD) is a chronic inflammatory skin condition that can affect the quality of life with frequent recurrences. There is no medication as yet to cure this disease completely. There are four general categories of agents that are used to treat SD: antifungal agents, keratolytics, corticosteroids, and lastly calcineurin inhibitors. Topical therapies are the mainstream line of treatment to be used for this skin condition.

The objective of this article is to critically review the published data in the literature on the use of topical pimecrolimus 1% topical cream as an option for treating facial SD. The final purpose of this review is to answer two questions: whether pimecrolimus topical cream is effective for the treatment of SD compared to the conventional current treatments and how safe is this treatment.

The PubMed, Clinicaltrials.gov, MEDLINE + Embase, and Cochrane library databases were searched for original randomized clinical trials (RCTs) evaluating pimecrolimus 1% topical cream and comparing it with other topical treatments for SD. A systematic review and meta-analysis were then conducted on the selected studies by grading the evidence and qualitative comparison of results among and within studies. A total of five studies were included in the review; however, only four were eligible for inclusion in the meta-analysis, in which pimecrolimus was compared with other treatments for the management of facial SD.

Pimecrolimus was found to be an effective topical treatment for facial SD, as it showed considerable desirable control of the symptoms in patients with facial SD clinically, in addition to a lower recurrence or relapsing rates; however, it had more side effects compared to other topical treatments, but the side effects were mild and tolerable.

## Introduction and background

Definition

Seborrheic dermatitis (SD) is described as a chronic inflammatory skin condition distinguished by exacerbation and remission in immunocompetent adult patients. Within the overall adult population, SD can be found in 1-5%, with no specific predilection to a specific ethnicity [1]. A more transient infantile type of SD presents and later quickly resolves within the first three to four months, while there are other cases that are more persistent and could reoccur over the span of months. Infantile SD could be diffuse or limited to the scalp (the so-called “cradle-cap”). The adult type is way more common than infantile SD, and appears to occur more frequently in men (3%) as compared to women (2.6%) across all age groups [2-4]. Adult SD may appear initially around puberty, correlated to the increase in cutaneous lipids via sebum secretion androgen-driven sebaceous gland development [4]. The trajectory of adult SD in affected individuals varies from individual to individual throughout the course of adulthood. Some patients will only experience occasional periods of exacerbation, whereas others may have greater chronicity due to more frequent recurrences [3].

Immunocompromised individuals such as patients with acquired immunodeficiency syndrome (AIDS) are disproportionality affected by SD. The incidence of SD in patients with AIDS is estimated to be between 30-80%; this likely correlates with T-cell lymphopenia, and the associated drop in CD4+ cell count hindering active immune surveillance [4].

Epidemiology

Seborrheic dermatitis has a biphasic incidence, occurring in infants between the ages of two weeks and 12 months and, later, during adolescence and adulthood. The prevalence of clinically significant seborrheic eczema is thought to be around 3%; however, data from retrospective and cross-sectional studies report a prevalence of 2-8%. The prevalence is likely to be much higher when mild cases are considered with prospective studies reporting figures as high as 23% in selected populations [3,5]. The Rotterdam Study challenges these figures and suggests a point-prevalence of 14.3% and the authors believe that their data reflect true prevalence as their study is population-based and accounts for all cases not just moderate to severe cases captured from health records used in previous studies [5]. The peak prevalence occurs within the third and fourth decades of life, although cases have been reported after the age of 50 [3].

The male preponderance of SD might be explained by the fact that females tend to be more careful when selecting skin and hair products, and usually take better care of their scalps and self-hygiene. Self-reporting bias may also occur as denser hair on the female scalp may conceal the clear picture of dandruff and milder forms of SD [6]. Another possible explanation is the different levels of sex-related hormones between men and women. This observation is based on the fact that SD incidence peaks during the hormonal surge of puberty. This theory, however, is not supported by evidence as logistic regression analyses from Rotterdam Study failed to establish an association between baseline hormone levels and the presence of SD among study subjects [5]. 

The prevalence of SD is increased among individuals with HIV infection, in whom it might be a presenting sign. The prevalence has been estimated to be around 35% among patients with early HIV infection and up to 85% among patients with AIDS [7,8].

Patients with parkinsonism frequently present with seborrhea (oily skin) and SD, both of which can improve with levodopa therapy [9].

The burden of the disease

SD is characterized by a pattern of both relapsing and remitting. In regards for its potential to affect the quality of life (QOL), it ranks third after atopic and contact dermatitis [10].

An estimated 50 million Americans experience dandruff, contributing to spending 300 million dollars annually purchasing over-the-counter products to help soothe and treat scalp itching and flaking. Not only do these individuals suffer symptoms of physical discomfort such as itching, dandruff negatively impacts patients’ self-esteem and is deemed socially embarrassing [6].

Albeit SD is a much less common case, in the United States, its outpatient office visits alone cost 58 million dollars in 2004, with a massive 109 million dollars spent on its prescribed medications. Over-the-counter products along with hospital services combined, there is an estimate overall direct cost of SD amounting up to 179 million dollars, with another 51 million dollars to make up for indirect costs, such as absent working days. One should also bear in mind that given the likeliness of SD presenting itself on visible areas of the patient, such as the face, there remains a negative impact on the individual’s QOL, presented in the form of low self-esteem or psychological distress; to relieve symptoms, patients were collectively willing to pay 1.2 billion dollars yearly. While the QOL in SD patients ranks lower than in those patients experiencing atopic or contact dermatitis, the QOL still remained higher than those with skin ulcers and solar radiation damage. In addition, the populations that are most affected are women, younger patients, and patients with higher educational levels [11].

Etiopathogenesis of SD

Role of Microflora and Fungal Colonization

Although the etiology of adult SD isn’t definitely known, there are three principal factors that appear to play a role: sebaceous gland secretion, alteration in colonization and metabolism of cutaneous microflora (*Malassezia *spp), and individual susceptibility and host response [12,13].

The role of *Malassezia *spp* *in the pathogenesis of adult SD remains controversial. However, the correlation between the use of ketoconazole shampoo, reduction in *Malassezia *spp, and the clinical improvement of scalp eczema have caused researchers to suspect that these commensal yeasts play a crucial role. Although the proliferation of *Malassezia *spp has been related to the exacerbation of SD, some reports have denied this association [14,15]. As *Malassezia *spp are not only present on the surface of the skin but also within layers of the stratum corneum, variations in technique in obtaining specimen and quantifying the organism likely elucidate the differences in findings among existing studies. The *Malassezia *spp that are most commonly associated with SD are *M. globosa* and *M. restricta*, both of which are commensal yeasts that need an exogenous source of lipids [16].

It has been suggested that *M. globosa* and *M. restricta* are capable of degrading lipids in sebum with the production of free fatty acids and triglycerides, followed by the consumption of certain saturated fatty acids. The remaining modified unsaturated short-chain fatty acids are more capable of penetrating the skin and inducing inflammation [3].

Immunocytochemistry and Biological Markers

The genetic components of SD and dandruff had been under-appreciated until recently when studies in animal models and humans identified inherited dominant and recessive types of SD and dandruff. In the autosomal recessive “inherited seborrheic dermatitis” (seb) mice, a spontaneous mutation within the outbred Him:OF1 mice caused seborrhea, rough coat, alopecia, growth retardation, and sometimes abnormal pigmentation in homozygous mutants [17]. Histological examination has shown enlarged sebaceous glands, hyperkeratosis, parakeratosis, acanthosis, and inflammatory infiltrates in the epidermis and dermis. Neither yeasts nor dermatophytes were detected. These mice were the primary animal model of SD to point out a clear mode of inheritance; however, the underlying mutation remains unidentified [18].

Consistent with a role for altered immunity in the pathogenesis of SD, transgenic mice carrying the 2C T-cell receptor (TCR) transgene in the DBA/2 background developed an extremely inflammatory phenotype in seborrheic areas, such as the ears, around the eyes, and muzzle area. Additionally, positive fungal staining by periodic acid-Schiff (PAS) was consistently detected in lesional skin but not readily apparent in non-lesional skin from diseased mice or from DBA/2 control mice. Furthermore, antifungal treatment reversed clinical and pathological presentations and reduced PAS staining [19]. These observations support the notion that the immune-compromised state and yeast infection play active roles in SD.

Host Responses

Another explanation suggests altered host immune function or response in those with SD. An augmented inflammatory response in lesional skin with an increase in natural killer cells (NK1+), CD16+ cells, inflammatory interleukins, and activation of complement cascade was observed when compared to non-lesional and healthy skin. The altered immune state in patients with AIDS, and the degree of immunosuppression, correlate with the progression and severity of SD, further solidifying the observed link between host response and the pathophysiology of seborrheic dermatitis [3,20].

Sebaceous Gland and Skin Barriers

The stratum corneum, the a-nucleated outermost skin layer, works as a bidirectional barrier that prevents both water loss and the entrance of microorganisms and environmental harmful agents [2]. It is composed of several layers of terminally differentiated keratinocytes, known as corneocytes. These unique keratinocytes are encased in lipid lamellae, bound together by desmosomes which are specialized intercellular cell adhesion structures. Any disruption within the lamellar lipid composition, corneocyte size or shape, desmosome number, and stratum corneum thickness, may lead to alterations in the epidermal permeability barrier (EPB) function [21].

Normally, sebum may change the intercellular lipid organization to assist desquamation. In SD and dandruff, however, altered corneodesmosomal hydrolysis may disrupt lipid organization and disturb the desquamation process, resulting in aberrant barrier function [22,23]. In support of this notion, barrier structural abnormalities are detected in dandruff scalp by electron microscopy that included intercellular *Malassezia* yeasts, changes in corneocyte shape and corneodesmosomes, and disrupted lipid lamellar structure. According to the structural findings, dandruff patients have been found to be more reactive (higher itch perception or flaking) than controls who were using topical applications of histamine or oleic acid to the scalp. These observations indicate that disrupted EPB function can contribute to the aggravation of dandruff [21,24]. Recent genetic studies done by Birnbaum et al. in humans and animals suggested that barrier dysfunction by itself can end up directly in SD-like conditions [25]. Biochemical studies and subsequent analysis have shown that the protein profiles in addition to those of SC ceramides and free fatty acids, have been changed and modified in patients with dandruff/SD, and most importantly, in the absence of apparent inflammation. These studies illustrate the significance of barrier restoration and maintenance in the management of SD and dandruff as well [26].

Neurogenic Factors and Emotional Stress

The high incidence of SD in patients with Parkinson’s disease and neuroleptic-induced parkinsonism has long been observed, especially in those with severe seborrhea, which provides favorable conditions for *Malassezia* proliferation. Studies suggested that SD is not purely neurological but more likely neuro-endocrinologically regulated because when patients with unilateral parkinsonism were observed, bilateral seborrhea was seen, and that goes in hand with the belief that it is more systematic than a localized disease [27-29]. In favor of this conclusion, α-melanocyte-stimulating hormone (α-MSH) levels in Parkinson’s patients were elevated, most likely due to inadequate dopaminergic input. Moreover, treating those patients with L-dopa resulted in a reduction of the levels of α-MSH, the re-synthesis of the MSH-inhibiting factor, and ending in the reduction of sebum secretion [29,30].

Additionally, there’s a piece of evidence for linking neurological damage; for example, patients with traumatic brain and spinal cord injury, and the incidence of SD [31]. Facial immobility of parkinsonian patients (mask-like face) and immobility caused by facial paralysis can induce elevated sebum accumulation and lead to SD, but only on the affected side [32,33]. Because poor hygiene has been implicated in SD, these observations suggested that sustained reservoirs of residual sebum related to immobility may influence the manifestation of the disease [6,34]. Additionally, SD is more commonly seen in depressive disorders and emotional stress [35].

Clinical picture and diagnosis

Clinical Diagnosis

The clinical presentations of SD in children and adults are often presented as well-delimited erythematous plaques with greasy-looking, yellowish scales of varying extents in regions rich in sebaceous glands, like the scalp, the retro-auricular area, face (nasolabial folds, upper lip, eyelids, and eyebrows), and the upper chest [35].

On the face, involvement of the glabella and malar regions, the nasolabial folds, and also the eyebrows are characteristic. Involvement of the eyelids may result in blepharitis. In men, the beard area may be affected by SD lesions. The scalp scaling associated with SD and dandruff is frequently bothersome as flakes, which are shed from the scalp and are often visibly apparent on darker clothing [36].

The distribution of the lesions is usually symmetrical, and SD is neither contagious nor fatal. SD shows a seasonal pattern; it is seen more frequently during winter and patients show improvement usually during summer [27,28,37]. Additionally, aggravation of SD symptoms has been associated with stress and sleep deprivation [38,39].

Histopathological Diagnosis

The dermatopathology of SD is non-specific, but the surface and infundibular epidermis usually show a superficial perivascular infiltrate of lymphocytes, acanthosis, focal spongiosis, and focal parakeratosis [12,36]. “Shoulder parakeratosis” refers to scale-crust accumulation surrounding the infundibular ostia. *Malassezia* can be present within the stratum corneum.

Histological progression from acute to chronic SD characteristically demonstrates a transition from spongiosis to psoriasiform hyperplasia and the development of a lichenoid lymphocytic infiltrate. Severe SD is usually related to keratinocyte necrosis, focal interface destruction, and leukocytoclasis [40].

Management of seborrheic dermatitis

The management plan depends mainly on two factors: (1) the patient’s age, and (2) the distribution and the severity of the condition. It’s essential to first counsel the patient about healthy general skincare practices, starting by encouraging the use of medical soaps and appropriate skin moisturizing [41]. Treatments should address the underlying disease process and any secondary features, especially the hyperkeratotic scale, bacterial (staphylococcal) infection, and associated symptoms, particularly pruritus [42]. 

The formulary used in SD includes antifungals, keratolytics, antipruritic, and anti-inflammatory agents such as topical corticosteroids and calcineurin inhibitors [43]. For scalp and non-scalp SD, current evidence supports the application of topical 1-2% ketoconazole, 1% ciclopirox, 1% zinc pyrithione, and 1% hydrocortisone [43,44]. Although potent corticosteroids are often necessary as a short-term remedy for scalp adult SD, the intermittent use of a mild topical corticosteroid in combination with antifungal agents such as imidazole is usually convenient and effective. There is an argument for using these agents in a rotatory manner to reduce the likelihood of adverse events and better efficacy when compared with monotherapy [43]. 

Shampoos usually contain combinations of agents like pine or coal tar (antipruritic/keratolytic), salicylic acid (keratolytic), sulfur (antimicrobial/keratolytic), and sulfacetamide (anti-inflammatory/antibacterial). The patient can apply these to the scalp and non-scalp regions and wash off after 5-10 minutes. Care should be taken when using topical salicylic acid, selenium, or zinc for the treatment of infantile SD, given the lack of safety and efficacy data to proceed with such treatment, but topical ketoconazole has been shown to be safe in infants with minimal systemic absorption detected [42]. 

A Danish expert group recommended guidelines and that practitioners should adopt topical antifungals as the first line of treatment, and agreed that topical corticosteroids and calcineurin inhibitors should only be used for more severe symptoms and to manage moderate to severe flare-ups [45]. In infantile SD, removing the scale-crust in the cradle cap in addition to reassuring the parents with their anxiety regarding their baby are essential considerations to be taken [46]. The cradle cap scales might be taken out using a soft-bristled toothbrush after the application of soothing cream or lotion such as Sorbolene. Another point that needs to be taken into consideration is that it is crucial to alleviate itching and discomfort in adult-type SD [42]. 

Side effects related to topical corticosteroids should be mitigated by intermittent use of site-appropriate potencies or steroid-sparing preparations like topical 1% pimecrolimus. Another strategy is to employ the inherent anti-inflammatory effect of the topical antifungals, estimated to be similar to 1% hydrocortisone [47]. Oral treatment should be considered for generalized or refractory disease, and the standard of care utilizes the antifungal and anti-inflammatory properties of ketoconazole (monitor liver function; black box warning), itraconazole (check for CYP450 drug interactions; can worsen heart failure), and fluconazole (adjust the dose per renal function). Itraconazole has the best anti-inflammatory effect, while oral terbinafine could be more effective than oral fluconazole in severe SD. Low-dose isotretinoin is non-inferior to topical standard of care but is usually related to significant mucocutaneous side effects [48].

Itraconazole is effective in controlling flares, averting relapses, and improving the overall quality of life in moderate to severe cases of adult SD while displaying a favorable safety profile [49]. However, the same cannot be said about infantile SD as high-quality safety and efficacy data are lacking. A specialist team review is often needed before starting treatment in these cases [50]. 

In HIV-AIDS, antiretroviral treatment frequently improves SD, and SD may improve with L-dopa therapy in Parkinson’s disease. Future therapies for SD could target improving skin barrier function or restoring the skin’s surface lipid composition [42,50].

Topical antifungals

Shampoos for Scalp Seborrheic Dermatitis

Ketoconazole: It is an imidazole fungicide agent with activity against* Malassezia* spp, with a direct anti-inflammatory property [4]. Two formulations are usually used, 1% and 2%; however, it was shown that ketoconazole 2% is more effective than ketoconazole 1% shampoo. A trial of 66patients done by** **Piérard-Franchimont et al.has demonstrated excellent results in 73% of those patients, compared to 45% improvement in patients who applied the 1% formulation [51]. The study has shown that both formulations have shown superiority over placebo in decreasing the relapse rates. The standard recommended frequency with ketoconazole 2% shampoo is twice weekly over a usual duration of four weeks. Intermittent use of ketoconazole 2% shampoo, such as once weekly, has been shown to be effective in preventing relapse of SD [52]. The favorable safety profile of ketoconazole 2% shampoo is supported by studies demonstrating negligible percutaneous absorption and low potential for irritancy or contact sensitivity.

Ciclopirox 1% Shampoo: Ciclopiroxolamine (ciclopirox) exhibits antifungal activity against *Malassezia* spp as well as other superficial fungi. Williams studied five double-blind, randomized, vehicle-controlled trials of ciclopirox 1%, including dose-response evaluation, and it showed the superiority compared to placebo in treating patients with SD [14]. Twice-weekly use appears to be optimal over a usual duration of a minimum of four weeks [53]. As similar as with ketoconazole 2% shampoo, the safety and tolerability of ciclopirox 1% shampoo are highly favorable [54].

Other Shampoos: Zinc pyrithione 1% and 2% shampoos and other shampoos such as selenium sulfide 2.5% shampoo have revealed significant efficacy for scalp SD as shown by multiple studies. One of these studies by Wolverton demonstrates that these two shampoos were the most effective over other applications [55]. Salicylic acid shampoos could be used for the adjunctive benefit to reduce scaling; however, their efficacy has not been well studied for adult scalp SD. Tar shampoos have also not been well studied for adult scalp SD; in addition to that, they can stain blond, white, or gray hair with a greenish or brown color. Additionally, selenium sulfide shampoo may cause residual odor, discolor hair, or create a sense of hair being oilier [55].

Practical Application

Ketoconazole 2% shampoo or ciclopirox 1% shampoo could also be effective as monotherapy in patients with mild-to-moderate scalp SD when used twice weekly over a minimum of four weeks. More frequent use isn't likely to afford additional benefits for most patients. In more severe cases, the additional use of a topical corticosteroid (TCS) at bedtime, either using a solution, foam, or spray, over the primary one to two weeks, is usually helpful in expediting the resolution of signs and symptoms, with the antifungal shampoo treatment continued over a minimum of four weeks of use. If a frequent relapse is problematic, either antifungal shampoo could also be used long-term once or twice per week [3].

Topical Anti-Fungal Leave-On Applications for Scalp SD

Ketoconazole 2% Foam: Other options for scalp SD are available; a leave-on foam formulation of ketoconazole 2% can be applied. However, studies showed that in more refractory cases, the additional usage of mid-to-high-potency topical corticosteroids with the antifungal foam will lead to a faster and greater benefit [3].

Topical Anti-Fungal Leave-On Applications for Non-Scalp SD

Ketoconazole 2% Cream or 2% Gel: Application of ketoconazole 2% cream twice daily over four weeks has been shown to favorably alter SD symptoms, including glabrous skin, albeit with slower onset of action when compared to topical corticosteroids. Ketoconazole 2% in gel or foam formulations may offer an advantage in hairy individuals, such as males with heavy central chest hair, in addition to the ease of use in sites such as the groin and axillae [4].

Ciclopirox 1% Cream: In a double-blind, vehicle-controlled trial of subjects with facial SD, ciclopirox 1% cream twice daily was significantly superior to vehicle cream after 28 days, followed by successful maintenance control over the subsequent 28 days with once-daily application [56].

Other Antifungals: Topical miconazole 2% cream has some evidence regarding the beneficial effect in treating facial SD; however, data is still lacking for terbinafine 1% solution and terbinafine 1% cream [4,14].

Topical corticosteroids

Although there are plenty of studies regarding the efficacy of topical corticosteroid usage and their obvious role in the treatment of psoriasis, it is obvious to the researchers that there has been a relatively noticeable absence of recently published data on the employment of TCS for SD [57]. Nevertheless, TCS is still considered to be the first- or second-line agents for SD, depending upon the severity of the disease. TCS is considered an effective mode of therapy in clearing the visible signs rapidly, in addition to relieving other associated symptoms, through the use of low to low-mid potency formulations. For scalp SD, many cases are related to greater severity of pruritus than with facial SD, warranting the necessity for initial treatment over the primary week or two with a higher potency TCS, followed by tapering in frequency over the subsequent one to two weeks. It has been noted that the relapse of SD occurs sooner and more frequently with the use of TCS when compared to antifungal agents and other nonsteroidal topical therapies [58]. As TCS is available in different formulations, the clinician may select the potency and vehicle of the TCS, depending on the severity and sites of involvement. In most cases, TCS use will likely be limited to a maximum of four weeks, as the response is typically rapid. In order to reduce the rate and frequency of the relapses, gradual tapering off TCS is still the usually recommended practice; however, data comparing it to abrupt discontinuation of these drugs has still not been formally studied [3].

Systemic treatment

Oral antifungal agents showed apparent successful outcomes in some refractory cases to topical treatments; therefore, they can be considered as an alternative option, especially when other treatment modalities fail. The evidence surrounding their use, however, is scarce and mainly derived from uncontrolled, low-quality studies. The few published meta-analyses and systematic reviews suffered from great heterogeneity impacting meaningful statistical analysis [59]. One published randomized controlled trial showed statistically significant improvement in controlling exacerbations of SD but was underpowered due to the small sample size and lack of multi-center design [60].

In an open-label study where patients were randomized to receive two doses of fluconazole (300 mg each) one week apart, or itraconazole 200 mg once daily for seven days. The main outcome was mycological eradication rate measured at 30 days. Fluconazole was superior with a 97% eradication rate compared to 80% in the itraconazole group. The failure rate was similar (23%) in both groups, two months after cessation of treatment [61]. In another open study, a 92% mycological eradication rate was achieved with a seven-day course of itraconazole 200mg orally [62]. Studies comparing single versus multiple dosing of fluconazole show conflicting results [63]. Mycological, or clinical response, rates within the range of 65-92% are reported after one dose of 400-450 mg of fluconazole [64].

Calcineurin inhibitors (CNI)

CNIs are drugs used primarily as immunosuppresses, especially in patients who underwent organ transplantation. They play a role in inhibiting phosphatase calcineurin, hence the name calcineurin inhibitors. Three drugs are known for this group namely cyclosporine, tacrolimus, and pimecrolimus [65].

Topical CNI showed good and beneficial outcomes when treating inflammatory skin disorders. Of those, topical immunomodulatory agents pimecrolimus and tacrolimus are developed to treat diseases of skin inflammation. These medications are structurally similar and can affect T-cell activation, a critical step in the inflammation process. The arrival of topical calcineurin inhibitors offers a vital substitute to corticosteroid therapy for several inflammatory dermatologic conditions, such as SD [66].

Tacrolimus

Tacrolimus is a macrolide derived from a bacteria called *Streptomyces tsukubaensis*. The drug was initially developed to be used in patients with graft organ rejection, but later became a favorable, safe, and effective topical anti-inflammatory agent. Tacrolimus’s mechanism of action is comparable particularly to that of pimecrolimus, given the formation of complexes composed of immunophilin macrophilin-12. The emergence of this complex inhibits inflammatory T-cell transcription. This tacrolimus-macrophilin-12 complex also has the ability to inhibit mast cell degranulation and the release of pro-inflammatory mediators. A notable distinction to consider comparing the immunophilin complexes accounted for by the two CNIs is that the tacrolimus-macrophilin-12 complex influences the differentiation of dendritic cells [67]. An in vitro study by Panhans-Gross et al. reported tacrolimus’s inhibition of the expression of the IL-2 receptor CD25, in addition to the co-stimulatory molecules, CD40 and CD80 [68]. However, tacrolimus had no effect on the expression of CD86. With the expression of Langerhans cell's surface molecules being altered, this may lead to a decrease in their stimulatory activity. Additionally, tacrolimus is not only limited to showing anti-inflammatory activity but also possesses activity against fungal strains, including *M. furfur* and various other *Malassezia* strains. Tacrolimus also demonstrates an enhanced function in the epidermal barrier, a beneficial property considering its disruption due to the pathogenesis of SD. The improvement was sustained after the treatment was stopped per a study byXhauflaire-Uhoda et al. [69].

Pimecrolimus

Pimecrolimus is known as an ascomycin macrolactam. Several in vitro studies exhibit pimecrolimus’s inhibition of the transcription and proinflammatory cytokine release in T-cells, owing to its cell-selective mechanism of action [70]. Binding to immunophilin macrophilin-12 (FK-506 binding protein), pimecrolimus makes a complex with considerable effects on the immune system. Stopping de-phosphorylation is the first and main effect of the pimecrolimus-macrophilin-12 complex, therefore, affecting the nuclear factor of activated T cells (NF-AT) cytoplasmic component via the Ca2+/calmodulin-dependent protein phosphatase, halting the inflammatory process [71].

Only by being dephosphorylated can NF-AT be translocated within the cell’s nucleus and therefore initiate the inflammatory cytokines’ synthesis. The flow of effects due to the pimecrolimus-macrophilin complex on NF-AT ultimately leads to the inhibition of both IL-2 and IL-4 synthesis. Cytokines, such as IL-5, IL-10, and tumor necrosis factor-a (TNF-4), are also reduced in a dose-dependent manner. The immediate effect on the release of IL-2 is possibly due to down-regulation of IL2-receptor surface expression. Research performed by Grassberger et al. supports this theory and portrayed that greater pimecrolimus concentrations inhibit T-cell proliferation, regardless of the presence of exogenous IL-2 [72].

Another impressive effect of the pimecrolimus-macrophilin-12 complex’s inhibitory abilities is the prevention of pro-inflammatory mediator release from mast cells. The anti-inflammatory effects of pimecrolimus are a consequence of the inhibition of pre-formed inflammatory mediators, such as histamine, serotonin, and b-hexosaminidase [73,74].

Application site irritation was the most common side effect reported with the use of pimecrolimus 1% cream in some patients; however, most of those patients didn’t have significant problems with the application to the affected skin [70]. A mild burning sensation soon after starting treatment was the second most common adverse effect reported by patients. About 10-37% of patients in clinical trials of patients with SD treated with pimecrolimus 1% cream reported this, but at the same time, it almost always subsided within the first three days of therapy and most of the time it didn’t cause major problems for the affected patients [75]. It is worth mentioning that the rate of the burning sensation was lower in patients receiving topical hydrocortisone when compared to the topical pimecrolimus 1 % group [76].

Efficacy of topical pimecrolimus in the treatment of SD

Several studies have evaluated the efficacy of pimecrolimus for the treatment of SD. A large randomized, double-blind, vehicle-controlled, clinical trial by Warshaw et al. evaluated 96 patients with moderate-to-severe facial SD [77]. Patients were randomized to treatment with either pimecrolimus 1 % cream or a vehicle-controlled placebo twice daily for four weeks. The study showed that efficacy, measured by investigator global assessment, was greater for the pimecrolimus-treated group compared with the vehicle group at weeks two and four, irrespective of the severity of disease at baseline. In another study by Firooz et al., the efficacy of topical pimecrolimus in achieving complete clearance of SD lesions in 83% of patients after two weeks of twice-daily application was demonstrated [78]; however, Cook and Warshaw showed that there was no statistically significant difference between the numbers of patients achieving complete clearance after two weeks within the pimecrolimus group compared with the active control group treated with hydrocortisone 1% cream [70].

A single-center, open-label pilot study investigated the utilization of pimecrolimus cream in the treatment of 21 HIV-positive patients with mild-to-severe facial SD [79]. Researchers found a big improvement in erythema, scaling, and infiltration/papulation by day seven of treatment, with over 90% of participants clear by two weeks. Patients also rated a significant improvement in erythema, scaling, and pruritus. All patients had a good effect of the therapy, despite varying degrees of immunodeficiency, with no effect on patients’ CD4 or CD8 T-cell counts or viral load.

Rallis et al. confirmed the efficacy of pimecrolimus cream in an open-label, non-controlled study of 19 adult patients with SD of the face and upper trunk [75]. Patients applied pimecrolimus cream twice daily for seven days, then continued application for an extra seven days if needed. After seven days of treatment with pimecrolimus 1% cream, 63% of patients had complete clearance of SD, with a mean overall severity score improvement of 83.7%. All study participants showed complete clearance of SD lesions at the end of week three.

Case reports of treatment with pimecrolimus 1% cream provide support for its efficacy in treating SD refractory to topical corticosteroids and ketoconazole. Crutchfield first documented the efficacy of pimecrolimus cream in the treatment of facial SD in a patient unresponsive to hydrocortisone 1% cream [80].

## Review

Aims and hypothesis

Facial SD is a chronic and difficult-to-treat condition with functional and psychological ramifications on patients’ wellbeing. Current conventional treatments, although relatively effective in controlling symptoms, come with various limitations that hamper their full potential. Pimecrolimus is emerging as a potential therapeutic alternative to overcome these limitations.

Although there is a decent body of evidence that establish topical calcineurin inhibitor as an effective and safe option in many skin conditions, evidence surrounding the specific use of pimecrolimus in facial SD is limited, and the largest systematic review and meta-analysis to date done by Ang-Tui et al. [81] is almost a decade old and was criticized for lack of pre-specified protocol, unclear exclusion criteria, lack of screening for publication bias, in addition to concerns regarding the validity of meta-analyses methods used [82].

The current study aims to evaluate the efficacy and safety of topical pimecrolimus in treating facial SD, through a well-executed systematic and meta-analytic review. The aim of this study is to answer the following questions: (i) What is the clinical effectiveness of pimecrolimus for the treatment of adult facial SD? How does it compare to other conventional therapies? (ii) How safe is pimecrolimus in patients with facial SD?

Research methods

Literature Search and Data Collection

For this systematic review and meta-analysis, an electronic search was conducted to identify randomized controlled trials for Pimecrolimus for the treatment of seborrheic dermatitis in accordance with PRISMA guidelines [83]. Broad search terms such as Pimecrolimus and Seborrheic Dermatitis were used to ensure that other potential studies concerning the intervention were not missed. Scientific literature was searched in the following databases: (1) PubMed, (2) Cochrane Library, (3) EMBASE, MEDLINE, and (4) Clinicaltrials.gov. In addition, a manual search was conducted by identifying a secondary reference list to screen for relevant randomized controlled trial studies written in English, from inception until August 1st, 2021.

Eligibility Criteria

Inclusion Criteria: The following were included: (i) studies with adult patients ( >= 18 years) diagnosed with facial SD, (ii) studies in which patients received pimecrolimus 1% topical cream as a single agent, (iii) placebo-controlled studies or those receiving other usual standards of care such as topical keratolytic, corticosteroids, or antifungal agents, (iv) studies that included short-term efficacy outcomes, and preferably, long-term and safety outcomes, if available, (v) studies that included short term outcomes such as subjective measures including improved symptoms like itching, burning, and objective dermatologist assessment including reported success rates, eczema area, and severity index and any other measures if comparable; for example, the percentage of body surface area (BSA) affected or the BSA%, (vi) studies with long term outcomes such as comparing the data found in the targeted studies with the main measure, that is the rate of recurrence, when applicable, (vi) studies including immediate, short, and long-term side effects, when comparable data is available, (vii) RCTs with any sample size, followed up for at least four weeks.

Exclusion Criteria: The following were excluded from our review: (i) studies with populations with facial dermatosis other than facial SD, (ii) studies with populations with any form of SD that does not involve the face, (iii) studies with populations who had severe SD and required systemic treatment, as this will likely render topical treatment redundant, (iv) studies that recruited HIV-positive participants or other malignant or viral infections requiring immune-suppressive therapy; SD is highly prevalent among immune-compromised patients [7,8] and immune-suppressive and anti-retroviral drugs used to treat these conditions were shown to improve SD symptoms [42,50], and including these studies would likely introduce confounding bias to the results, (v) studies with populations who had received antibiotics, immunosuppressive drugs, or phototherapy one month prior, and/or any topical therapy suspected to affect SD during the week preceding the study were excluded to adjust for residual confounding, (vi) studies in which patients did not receive topical pimecrolimus 1% cream in the intervention arm, or those who have received any form of combined active treatment with pimecrolimus, (vii) studies with comparator groups who have received any form of treatment other than topical preparations, such as those who received oral medications, phototherapy, or parenteral agents were excluded, (viii) studies that did not include short-term efficacy outcomes, (ix) all non-randomized trials including case series, case reports, cross-sectional studies, or observational studies were excluded, (x) unpublished trials or studies reported only as conference abstracts, (xi) trials not published in English; despite the inherent risk of bias when excluding non-English studies, in reality, there are no significant changes in the outcomes of the systematic reviews when language is restricted to English only, according to the Cochrane Handbook [84].

Search Strategy

The search query was generated to suit each electronic database used in data collection, and the advanced search field was used when applicable to identify Participants (P) and Intervention (I) to generate the initial search query using “EXPLODED” Medical Subject Headings (MeSH) terms. Then, appropriate filters (Randomised Control Trials, and Clinicaltrials.gov) were applied to generate results relevant to the subject. Each database used required a unique search string, as below:

(((((Dermatitis, Seborrheic[MeSH Terms]) OR (Seborrheic dermatitis[MeSH Terms])) OR (Seborrheic Eczema[MeSH Terms]))) AND (Pimecrolimus[MeSH Terms])) OR (Elidel[MeSH Terms])

Searching PubMed and applying the MeSH terms, a total of 172 studies were generated. By using the SAVE option on the website, all the results were chosen and then transferred and created into a new PubMed format, which then was downloaded into a computer file and then uploaded to a referencing management software (Rayyan, Rayyan Systems Inc., Cambridge, Massachusetts, United States) [85]. The same was applied when using Embase and Clinicaltrials.gov websites; the search terms used in PubMed were used here and resulted in 178 and 42 studies, respectively. Files were created through the export option, downloaded, and later uploaded directly into the online referencing management application. On the Cochrane Library website, the same terms were used and resulted in a total of 531 results; however, for the last two years, Cochrane has not allowed the citation exporting function, as it became available for the users who hold membership at their site, therefore, the studies were manually exported and reflected into a text file that supports BibTex, which was reflected the aforementioned application (Rayyan) [85].

All eligible studies were manually reviewed by examining the titles and abstracts first to remove duplicates and irrelevant studies, then the full text was scanned and compared to the proposed eligibility criteria to illuminate irrelevant studies, then the final list was pooled for an in-depth review of the individual studies.

Data Extraction

Data extracted included the following summary data: (i) the population characteristics as age, sex, symptoms of seborrheic dermatitis including itching, burning sensation, and scaling, in addition to disease severity, (ii) sample size and distribution, (iii) The intervention arm of topical pimecrolimus 1%, duration of the treatment and the dosage, (iv) control arm (placebo or comparator topical agents including topical anti-fungal agent and different forms of topical corticosteroids), (v) primary outcomes, (vi) adverse effects, and (vii) follow-up duration and timescales to reflect disorder state. The severity of the disease was assessed clinically and scored accordingly as a baseline and through the follow-up periods. The outcomes were represented in the studies and reported as clinical improvements of the symptoms in addition to the duration needed for those symptomatic features to show improvement. Lastly, the adverse effects of the treatment, whether it was on the intervention arm or on the control arm, were reported and also followed up.

Risk of Bias in Individual Studies

The risk of bias for each included trial (four trials) was independently assessed by Version 2 of the Cochrane risk-of-bias tool for randomized trials (RoB 2) across the following domains: (i) Random sequence generation, (ii) Allocation concealment, (iii) Blinding of participants and personnel, (iv) Blinding of outcome assessment, (v) Incomplete outcome data, (vi) Selective reporting, and (7) Other sources of bias. The studies were rated as low risk, with some concerns or a high risk of bias [16]. The visual visualization of the assessment was achieved by the Risk-of-Bias VISualization (robvis) tool, an R-package, and Shiny web app (R Foundation for Statistical Computing, Vienna, Austria) [86], and supported by the National Institute of Health Research, Bristol, and funded by the National Institute for Health Research (NIHR). The tool is used to easily visualize the risk of bias assessments and produce high-quality summaries ready for publication [86]. The formatting involved manual downloading an Excel (Microsoft Corporation, Redmond, Washington, United States) template file and formatting the results according to the risk-of-bias assessment tool used to perform the assessments (RoB 2), and then uploading the Excel file to the web app to produce the results. 

Assessment of Publication Bias

Publication bias was subjectively assessed using funnel plots by looking for asymmetry in the study risk ratio (RR) against the standard error of the logarithm of the RR. The objective evidence of bias that used Egger’s regression asymmetry test was not applied, as the number of studies was small.

Data Management

Records were managed through MyBib, which is a free online bibliography manager and citation generator or other specific software for managing bibliographies.

Data Analysis

In the analysis of the data, each of the eligible trials was assessed for risk of bias using risk-of-bias assessment methods, then evaluated separately by the difference in mean and standard deviation from the baseline to the end of the trial. The data were extracted against the two primary endpoints of meta-analysis: the efficacy of the treatment through clinical evaluation and the scoring of the SD symptoms, hence the improvement in symptoms of SD and the safety endpoints, and those were analyzed (when available); therefore, side effects of the treatments of both arms were analyzed in addition to the relapse rates and recurrences after stopping the treatments. Then the data were examined for heterogeneity to assess its impact on the meta-analysis, which was measured using the chi-squared test, and expressed between the trials through forest plots. Lastly, the data from all the trials were gathered and compared to reach the final results.

Assessment of Heterogeneity

Heterogeneity is calculated using the *I2* test, available in numerous statistical packages such as SPSS, and can now be even generated along with the forest plot, with the advent of Cochrane’s Review Manager (RevMan) Version 5.4 (The Cochrane Collaboration, 2020).

The four studies included in this meta-analysis were expected to have a high heterogeneity, which can be understood given the variation in methodology assessment tools and outcomes used in them. The primary endpoints of these studies relied heavily on subjective outcomes, which tend to lead to increased heterogeneity [87]. Some researchers would undergo subgroup analysis or meta-regression; however, for the purpose of this meta-analysis, a random-effect model was chosen as the heterogeneity cannot be explained by chance or solved by a different type of analysis [84].

Results

**Figure 1:**
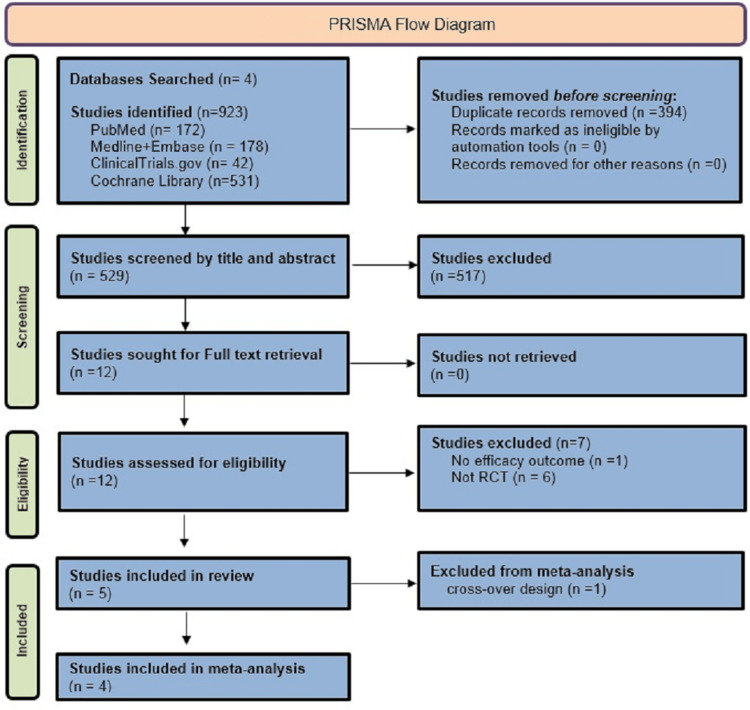
PRISMA chart PRISMA: Preferred Reporting Items for Systematic Reviews and Meta-Analyses

By searching the database, 923 trials were identified (172 trials were found in Pubmed, 178 in Medline and Embase, 42 in Clinicaltrials.gov, and 531 trials in Cochrane library). After removing the duplicates and applying the inclusion and exclusion criteria, five studies were included in the systematic review and from these five trials, only four were included in the meta-analysis. The search results are expressed via the Preferred Reporting Items for Systematic Reviews and Meta-Analyses (PRISMA) flow diagram (Figure 1).

**Figure 2:**
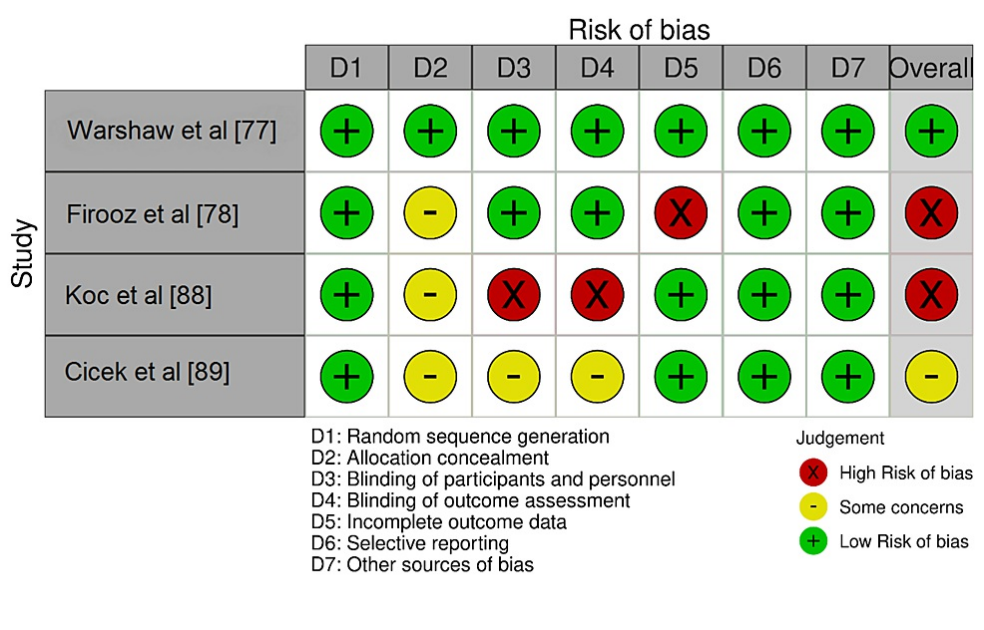
Risk-of-bias traffic light plot

**Figure 3:**
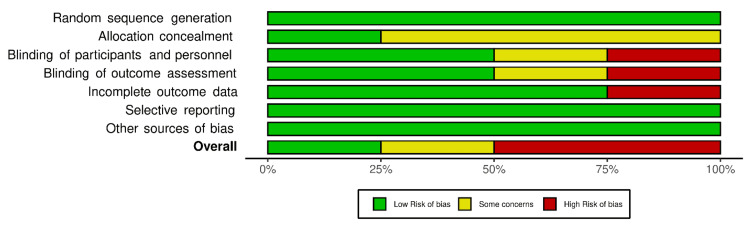
Risk-of-bias summary plot

The risk of bias of the four included trials in the meta-analysis was assessed using the RoB 2 and it is illustrated in Figures 2, 3. It was found that only the study by Warshaw et al. [77] had a low risk of bias. Two trials were considered an overall high risk of bias. Koc et al. [88] lacked participants and outcome assessment blinding and Firooz et al. [78] had a high risk of bias in the incomplete outcome data domain, while the study by Cicek et al. [89] was considered as having some concerns in domains 2, 3, and 4. 

Five RCTs were studied regarding the efficacy and safety of using pimecrolimus 1% topical cream for facial SD. One study, (Rigopoulos et al., 2004) showed that most of the participants had complete recovery, and all of them improved in less than a two-week period [77]. The study was a cross-over trial and it was done for only nine days; therefore, it was excluded from the meta-analysis, as it did not meet the eligibility criteria of the minimum of two weeks’ duration of the pimecrolimus 1% application. The remaining four studies were included as they fulfilled the criteria needed for the review. The characteristics of the included studies are summarized in Table 1. All the studies are more than a decade old with a total of 248 participants, 75% of which were males, and the topical treatments were used for at least two weeks.

**Table 1:** Characteristics of the included studies SD: seborrheic dermatitis; M: male; F: female

Author	Participants			Disease Definition	Intervention Arm (Pimecrolimus)				Control Arm							
	N	M	F		N	Drop out N %	Mean Age (Yrs)	Mean Disease Duration (Mo)	Control Drug	N	Drop out (N%)	Mean Age (Yrs)	Mean Disease Duration (Mo)	Frequency	Duration (weeks)	Follow-up (weeks)
Koc et al. [88]	48	42	6	Mild to Moderate SD	23	2 (8.6%)	32.3± 9.5	26.4	Ketoconazole	25	3 (12%)	29.8 ± 9.0	25.2	Twice Daily	6	0,2,6,12
Warshaw et al. [77]	96	85	11	Mild to moderate SD	47	0 (0%)	27 -84	137.9 ± 150.9	Vehicle	29	2 (4%)	20-88	134.9 ± 156.5	Twice Daily	4	0,2,4
Firooz et al. [78]	40	28	12	Adult Facial SD	20	2 (10%)	28.65 ± 7.75	46.05 ± 56.47	Hydrocortisone	20	1 (5%)	29.55 ± 7.44	37.45 ± 55.04	Twice Daily	2	0,2,4
Cicek et al.(a) [89]	64	32	32	Adult Facial SD	21	0 (0%)	31.6 ± 8.27	25.08 ± 9.96	Metronidazole	21	4 (19%)	30.7 ± 7.35	36.0 ± 36.96	Twice Daily	8	0,2,4,8
Cicek et al.(b) [89]									Methylprednisolone	22	0 (0%)	34.2 ± 15.69	27.72 ± 16.20	Twice Daily	8	0,2,4,8
	248	187	61		111	4 (3.2%)				117	10 (7.9%)				4.8	

The first study was a randomized, investigator-blinded trial, conducted in Iran by Firooz et al. in 2006 [78]. It compared hydrocortisone acetate 1% cream versus pimecrolimus 1% cream, in which both groups had similar outcomes in improving the symptoms of SD with no statistical significance showing that pimecrolimus could be a good treatment alternative. However, the pimecrolimus 1% group showed a higher rate of adverse events in form of a burning sensation (seven participants compared to one in the hydrocortisone group).

The next trial was done by Warshaw and his colleagues in 2007 in the United States [77]. This was a double-blinded trial in which pimecrolimus 1% was compared with the vehicle and reported that topical pimecrolimus 1% cream can be used as an effective treatment in controlling the symptoms of facial SD and, at the same time, it was tolerable with minimal side effects of transient tingling and burning sensation [77].

The last two studies were both conducted in Turkey in 2009 [88,89]. Koc and colleagues [88] ran an open, randomized trial for 48 participants, comparing pimecrolimus 1% cream (23) with ketoconazole 2% cream (25) showing that both treatments were comparable in their efficacy with no statistical significance at the end of the follow-up period with most of the participants of both groups showing significant improvement in their symptoms (erythema, scaling, and infiltration). However, ketoconazole 2% group showed more tolerability than the pimecrolimus group, as the latter have shown more frequent adverse effects (burning sensation, erythema, pruritus, and irritation); 19 patients in the pimecrolimus group compared to five patients in the antifungal group [88]. Cicek and his group [89], in their RCT, compared pimecrolimus 1% cream with two other topical applications, methylprednisolone aceponate 1% cream and metronidazole 0.75% gel, with 22 participants in the methylprednisolone group and 21 participants each in the pimecrolimus and metronidazole groups. The clinical severity score was almost similar for all the groups enrolled in the study at baseline. It measured the erythema, pruritus, and scaling for each participant, and then measured every two weeks for a total of eight weeks. This study illustrated that the three models of treatment used in the patients with SD were effective in decreasing and controlling the symptoms. It was found that pimecrolimus 1% topical cream showed a statistically significant difference when compared with the group using the topical corticosteroid and the group using the antifungal gel as well, and that was measured in week four, when the three groups were scored again according to the clinical severity score, after comparing it with the score measured at baseline. The adverse effects in this study for the group who used the topical metronidazole gel were the highest, in which eight out of the 17 patients who completed the trial have shown erythema, edema, a burning sensation, or itching as side effects of the treatment, whereas seven out of the pimecrolimus group showed burning sensation or erythema, and six out of the 22 patients who used the methylprednisolone have complained of erythema, edema or a burning sensation. However, regarding the adverse effects, the three modalities of treatment did not show statistical significance [89].

**Figure 4:**
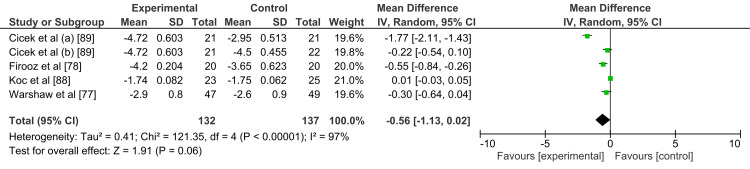
Forest plot showing the result of the efficacy of pimecrolimus SD: seborrheic dermatitis

The four trials reported the efficacy of pimecrolimus as the forest plot shows (Figure 4).

In the trial done by Warshaw and his colleagues [77], the confidence interval was between -0.64 to 0.04 while the mean difference was -0.30, in Firooz et al. [78], the confidence interval was between -0.84 to -0.26 while the mean difference was -0.55, in Koc et al. [88], the confidence interval was between -0.03 to 0.05 while the mean difference was 0.1. In Cicek et al [89], as two drugs were compared with pimecrolimus 1% topical cream, the results were separated into two groups; the first one was comparing the metronidazole gel with pimecrolimus cream and it showed the confidence interval was between -2.11 to - 1.43 while the mean difference was -1.77, and the second group compared methylprednisolone cream with pimecrolimus cream and it showed that the confidence interval was between -0.54 to 0.10 while the mean difference was -0.22.

The overall effect favors pimecrolimus over the control groups; however, the result was statistically insignificant (P=0.06). In addition, it was noticed visually from the forest plot that the heterogeneity of the trials was high and calculated via the I2 test as 97% (as it was explained earlier, the high heterogeneity was due to two reasons: firstly, the endpoints were based on subjective outcomes, and secondly, wide variations in methodology assessment tools and outcomes used in these studies).

Discussion

As time was of essence during the coronavirus disease 2019 (COVID-19) pandemic and timely findings were important for time-sensitive results, we decided to conduct a rapid systematic review. Due to time constraints inherent to the development of a rapid systematic review, no protocol for the systematic literature searches was registered beforehand with the International Prospective Register of Systematic Reviews (PROSPERO). This can be considered a limitation of this study. 

SD is a chronic, well-studied inflammatory skin disorder; however, for its chronic course and the difficulty of the current modality of treatment, in addition to the high recurrence rate, the need for a good, reliable, and tolerable new treatment is always required. From here, this study was designed to evaluate the efficacy and the safety of the CNI, pimecrolimus 1% topical cream, as it was assumed to be a good alternative for the conventional topical antifungal and corticosteroids.

The first point noticed during this analysis was the limited data and trials in the literature on the use of pimecrolimus as a possible treatment in SD, in addition to the outdated studies; the newest trial took place in 2009, which is more than a decade old, and new data from high-quality trials are required in this regard.

Another point noted was the high male-to-female ratios in all of the studies, which were three times greater in the male groups, and that strengthens the fact that SD is more reported in men than in women [7,80]. 

The most common symptoms in the patients who were enrolled in these trials were erythema and scaling, and these symptoms were included in the clinical scoring system used in those trials. Some trials included pruritus and others included infiltration in their scores and that made it more complicated to compare all of the symptoms in all the included studies.

Pimecrolimus 1% topical cream was shown to be effective or at least not inferior to the other treatments used for SD in all the trials done, and that can be noticed by the controllable symptoms by the use of this CNI topical application as compared to vehicle, hydrocortisone acetate 1% cream, ketoconazole 2% cream, methylprednisolone aceponate 1% cream, and metronidazole 0.75% gel. This provides strong evidence for the efficacy of this drug; however, there was no statistical significance to prove the superiority of any of the treatments used.

Tolerability for pimecrolimus 1% topical cream was only better when it was compared to metronidazole 0.75% gel and, otherwise, adverse effects were reported more frequently in the pimecrolimus group than in the other arm of the trials. However, it is worth noting that even though the side effects were more common when using pimecrolimus 1%, those symptoms were mild and very tolerable and rarely was treatment discontinued due to its adverse events. Those adverse events were a mild burning sensation, pruritus, irritation, and erythema and most of these symptoms were relieved after a few days after stopping the treatment. 

The follow-up period was short for most of the trials (except for one trial in which the follow-up period was up to 12 weeks). One trial followed the patients for eight weeks and the other two only followed their participants for four weeks, and this can’t be considered as enough time to estimate the efficacy of the medication and even more difficult to decide the recurrence rate after the cessation of the treatment. The relapse rate was shown in only two studies and in both, it favored pimecrolimus 1 % cream over methylprednisolone aceponate 1% and hydrocortisone acetate 1% creams, however, without statistical significance.

## Conclusions

Four RCTs comparing the efficacy of pimecrolimus 1% cream to topical corticosteroids and antifungals were identified. All showed that pimecrolimus 1% cream reduces the severity of SD with regard to scaling, erythema, and pruritus. However, adverse effects, although mild, were seen in a greater proportion of patients who applied pimecrolimus 1% cream. The relapse rate was also lower for pimecrolimus compared with topical corticosteroids. This suggests that pimecrolimus cream could be an effective alternative treatment for SD, especially for those resistant to topical corticosteroids and antifungals. Studies are required to further assess its dosing protocols and use as a long-term maintenance treatment to reduce relapses with the consideration of its safety profile.
